# Impact of *N*-Acyl-Homoserine Lactones, Quorum Sensing Molecules, on Gut Immunity

**DOI:** 10.3389/fimmu.2020.01827

**Published:** 2020-08-28

**Authors:** Garance Coquant, Jean-Pierre Grill, Philippe Seksik

**Affiliations:** ^1^Sorbonne Université, Inserm, Centre de Recherche Saint-Antoine, CRSA, Hôpital Saint Antoine, Paris, France; ^2^Department of Gastroenterology, Saint Antoine Hospital, Assistance Publique-Hôpitaux de Paris (APHP), Paris, France

**Keywords:** quorum sensing, gut microbiota, interkingdom communication, inflammatory bowel disease, gut inflammation

## Abstract

Among numerous molecules found in the gut ecosystem, quorum sensing (QS) molecules represent an overlooked part that warrants highlighting. QS relies on the release of small molecules (auto-inducers) by bacteria that accumulate in the environment depending on bacterial cell density. These molecules not only are sensed by the microbial community but also interact with host cells and contribute to gut homeostasis. It therefore appears entirely appropriate to highlight the role of these molecules on the immune system in dysbiosis-associated inflammatory conditions where the bacterial populations are imbalanced. Here, we intent to focus on one of the most studied QS molecule family, namely, the type I auto-inducers represented by *N-*acyl-homoserine lactones (AHL). First described in pathogens such as *Pseudomonas aeruginosa*, these molecules have also been found in commensals and have been recently described within the complex microbial communities of the mammalian intestinal tract. In this mini-review, we will expound on this emergent field of research. We will first recall evidence on AHL structure, synthesis, receptors, and functions regarding interbacterial communication. Then, we will discuss their interactions with the host and particularly with agents of the innate and adaptive gut mucosa immunity. This will reveal how this new set of molecules, driven by microbial imbalance, can interact with inflammation pathways and could be a potential target in inflammatory bowel disease (IBD). The discovery of the general impact of these compounds on the detection of the bacterial quorum and on the dynamic and immune responses of eukaryotic cells opens up a new field of pathophysiology.

## Introduction

Dysbiosis in inflammatory bowel disease (IBD) is characterized by a reduction in bacterial biodiversity. This change in biodiversity is associated with a reduction in the bacterial load of various bacterial groups and expansion of others (1). Bacteria are capable of exchanging small signaling molecules depending on the bacterial density, a process called quorum sensing (QS). QS coordinates gene expression and physiology of bacterial populations. There are several families of molecule used by microbes to communicate. A universal system relying on type II auto-inducers (AI-2) can be used by all bacteria (2). Gram-positive bacteria use oligopeptides, although Gram-negative QS display various molecules (3). Among them, the most studied system is represented by the type I auto-inducers based on *N*-Acyl-homoserines lactones (AHL). Beyond bacteria–bacteria communication, QS molecules are also involved in interkingdom interplay between gut bacteria and host cells. In the early 2000s, researchers investigated the impact of AHL on plant physiology (4) showing that the presence of these molecules produced by soil bacteria can induce plant benefits (5) such as induction of the immune system to resist pathogens (6, 7). More recently, underlying potential on health and disease, AHL have been described in mammals' gut lumen and lately in humans. It is likely that IBD dysbiosis is a condition that cannot only modify QS but also be maintained by QS. Therefore, a better knowledge of this largely overlooked metabolite component appears important to improve our understanding of the molecular mechanisms implicated in the immune gut responses in IBD.

### *N*-Acyl-Homoserine Lactones

#### Quorum Sensing Molecules for Bacteria

Bacteria are not single cells living independently. They have “social” interactions called QS. QS was first studied in the 1970s through the bioluminescence emitted by a marine bacterium, *Vibrio fischeri*. These bacteria live in symbiosis with marine animals. *V. fischeri* does not emit light at low concentrations in the water; however, when the bacterial population is growing in symbiosis with a squid, within a rich environment, the bacteria emits light (8). This phenomenon is possible, thanks to QS. However, the term was used for the first time two decades later, by Fuqua et al. (9).

**Figure 1:**
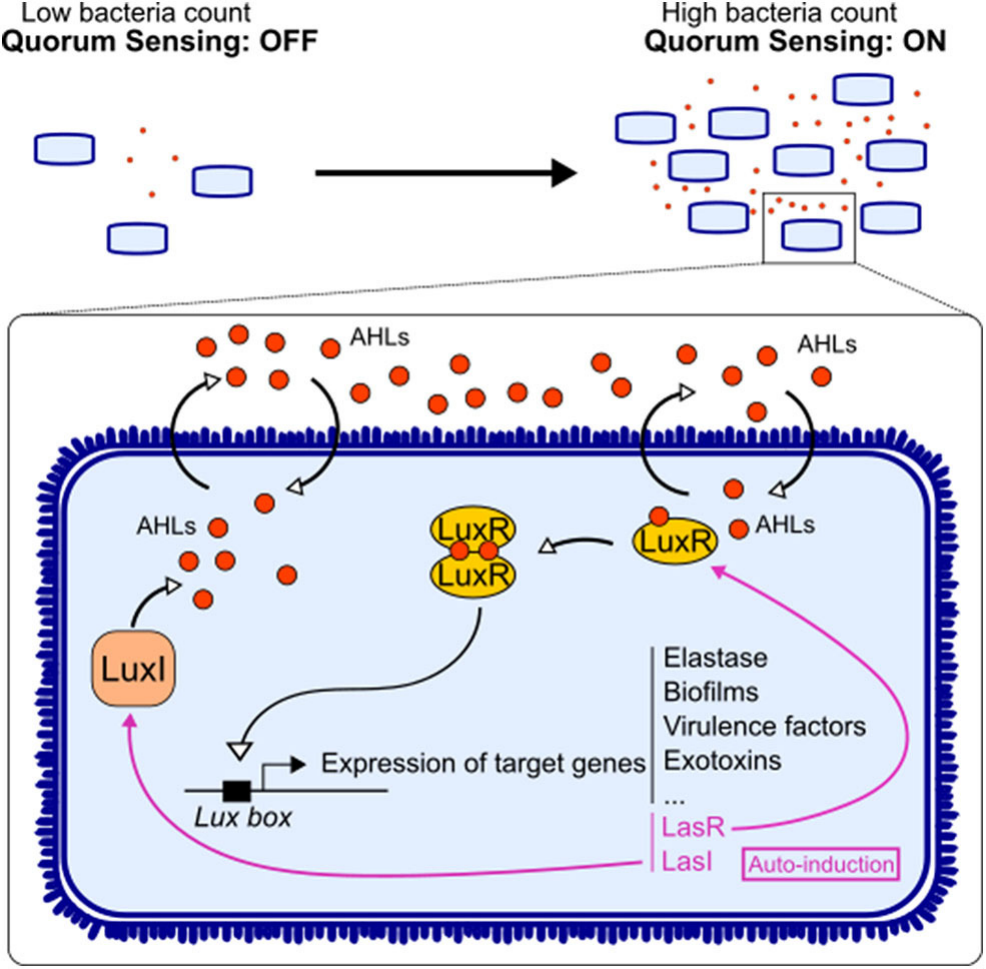
Quorum sensing signaling in bacteria. Acyl-homomserine lactones (AHLs) are auto-inducers used by Gram-negative bacteria to communicate. The enzyme LuxI synthesizes the AHL, and the latter can diffuse freely through the membrane. Upon reaching a threshold concentration, AHL can bind to its receptor LuxR. The dimerization of the receptor allows it to act as a transcription factor on the *Lux box*. This triggers not only the expression of target genes involved in the virulence of the bacteria but also the expression of AHL system LuxI/LuxR.

This system relies on the release, by bacteria, of small molecules (auto-inducers) that accumulate in the environment depending on bacterial density and are sensed by the bacteria community. Once a threshold concentration is reached, this triggers the expression of certain bacterial genes, encoding for virulence factors, biofilm formation, etc. (Figure 1). Gram-negative bacteria use AHL as one of the QS molecules (10). AHL is a family of molecules composed by a unique lactone ring, an acyl chain of various lengths, and substitutions at the acyl C3 position that define the molecular species. The integrity of the lactone ring is important for AHL immunoactivity (11). Quorum quenching is the mechanisms by which QS signal is degraded, and it can occur enzymatically, through acylases (cleavage of the AHL amid bond, releasing a fatty acid and homoserine lactone) and lactonases (hydrolysis of the HSL ring) (12).

#### AHL Genes Induction

AHL diffuse freely in bacteria and recognize the receptor LuxR, allowing them to act as a transcription factor. Once activated, QS induces the expression of a series of bacterial genes contributing to bacterial virulence and/or adaptation such as toxins, motility, enzymes, secretion systems, iron uptake, metabolism, and biofilms (Figure 1) (3). AHL also regulate their own synthase and receptor genes, in a positive retro-control loop. This system has been mostly described in pathogens, while the impact of these factors on gut immunity has not been directly investigated. However, few reports showed that AHL found in gut ecosystem could benefit host physiology (13). Thus, there is still a gap in research on AHL derived from commensals and their impact on host.

Most studies have focused on the impact of auto-inducers from the opportunistic bacterium *Pseudomonas aeruginosa, N*-(3-oxododecanoyl)-l-homoserine lactone (3-oxo-C12-HSL) and *N*-butyryl-homoserine lactone (C4-HSL). Signaling works through the LuxI/LuxR homolog system. LuxI synthesizes the AHL, the molecule freely diffuses in the environment, and LuxR detects the auto-inducer, which acts as a transcription factor (14) (Figure 1). Bacteria can use different ways to communicate: (i) crosstalk, different species talking to each other; (ii) self-talk, bacteria from the same species talk with its own auto-inducer and regulate their gene expression; and (iii) eavesdropping, when bacteria from one species can intercept the signal of another species without creating a signal by itself (15). Indeed, some bacteria express a LuxR homolog without its partner LuxI. LuxR is then labeled LuxR solo, and the receptor is called SdiA (16). For instance, *Escherichia coli* has the SdiA receptor without being able to produce an auto-inducer (17). This ability to “listen” to other bacterial species is essential in complex ecosystem like the gut microbiota, where hundreds of different bacteria coexist. However, these ways of communicating bypass the bacterial world, and those small molecules are part of an interkingdom signaling, allowing cross-talk between gut microorganisms and the host.

#### AHL Interkingdom Signaling

Since eukaryotic and prokaryotic cells have coevolved for thousands of years, the signals from one have adapted to the other's. In this setting, bacterial QS molecules have been shown to have an effect on eukaryotic cells, a phenomenon called “interkingdom signaling” (18). To date, most studies on the effects of AHL on mammal cells have focused not only on the well-known 3-oxo-C12-HSL and C4-HSL from *P. aeruginosa* but also on *N*-(3-oxohexanoyl)-l-homoserine lactone (3-oxo-C6-HSL) from *V. fischeri*. AHL are amphiphilic molecules, as lactone ring remains hydrophilic and the acyl chain is hydrophobic. It has been shown that these properties allow the 3-oxo-C12-HSL to diffuse freely through the cell membrane of eukaryotic cells, similar to bacteria, while long-chained versions are mostly actively transported (19). AHL are chemically analogous to many lipid-based hormones such as the eicosanoid family of lipidic and steroid hormones involved in hundreds of biological functions in eukaryote. It is also thought that AHL can enter the host cell, bind to intracellular receptors, and regulate gene transcription (18). Using labeled molecules, it has been shown that, depending on cell lines, they can indeed enter cells and be detected in the nucleus or the cytoplasm (19, 20).

The AHL perception mechanism in mammalian cells is still fairly unclear. To date, no mammalian receptor has been clearly demonstrated; only certain hypotheses have been proposed. It has been shown that the AHL were not recognized by the classical pattern recognition receptors (PRRs) from the innate immune system like other microbial molecules (21). The studies done on the 3-oxo-C12-HSL produced by *P. aeruginosa* have identified different receptors based on the cell types and methods used. Proinflammatory effects have been associated with receptors such as nuclear factor kappa B (NF-κB) or activator protein-2 (AP-2) (21). Using other cellular models, mitogen-activated protein kinases (MAPKs) have been proposed as potential receptors (22). In 2008, Jahoor et al. identified the peroxisome proliferator-activated (PPAR) receptors PPARß/δ and PPARγ as potential AHL receptors (23). The interaction between 3-oxo-C12-HSL and PPARγ has also been reported by another group, at very low concentration (1 nM) of AHL (24).

Other candidate mammalian AHL receptors are the G-protein-coupled receptors and, among these, member 38 of the bitter taste receptor family (T2R38), one of the most studied bitter taste receptors. It is widely expressed in the human digestive tract from the tongue to the colon (25). In the lower gastrointestinal tract, T2R38 is suspected to play a role in eliciting immune responses to toxic compounds or pathogens in digestive diseases and metabolic conditions. Indeed, *TAS2R38* polymorphisms have been linked to increased susceptibility to infections and colorectal cancer (26–28). Moreover, reports shows that both 3-oxo-C12-HSL and C4-HSL can activate T2R38 in pulmonary epithelium (28). The interaction between AHL and this receptor was described in neutrophils, by immunofluorescence and pull-down assays (29, 30).

Another potential AHL receptor is the IQ-motif-containing GTPase-activating protein (IQGAP1) (31). IQGAP1 is a scaffolding protein, participating in cytoskeleton organization (32). It has been shown that IQGAP1 plays a role in tight junction assembly (32), and as 3-oxo-C12-HSL is known to disrupt junction integrity (10), the interaction between IQGAP1 and AHL seems rational.

Finally, according to the cell type, the receptors and their localization can be different (33, 34) but mainly involves inflammatory pathways. Host cells have developed the abilities to disrupt QS signaling, by degrading them through the production of paraoxonases (PON), which can be expressed by intestinal epithelial cells and macrophages (35). Regulation of this communication between host cells and bacteria can occur in gut ecosystem. Moreover, it has been shown that PON1 polymorphisms may confer protection against the development of IBD (36). Therefore, AHL interkingdom signaling can be viewed as potent axis of the gut microbiota–host crosstalk in chronic inflammation and especially in IBD.

### Impact of AHL on Innate Immunity

It has been shown, on cell lines and primary cell cultures, that AHL, depending on their acyl chain length, double bounds, and concentrations, may differently affect innate immune system.

Epithelial cells provide a physical barrier between the host and the lumen of the intestine, relying on tight junctions. This prevents the luminal content to harm the host's integrity. Epithelial cells contribute to the innate immune response, as they keep foreign particles from spreading into the host mucosa. Vikström et al. showed, on an intestinal epithelial cell line Caco-2, that *P. aeruginosa* 3-oxo-C12-HSL alters intestinal barrier, disrupting protein junctions integrity (37–40). When exposed to the AHL, permeability to macromolecules and ions is increased, and the expression and localization of junction proteins such as occludin, E-cadherin, and zonula occludens-1 is modified (37–39). This alteration process not only involves the MAPK, notably p38 and p42/44, but also needs the phosphorylation of junction proteins leading to disturbance of junction integrity (37–39). Alteration of calcium signaling is also part of the response to 3-oxo-C12-HSL (39, 41). Interestingly, C4-HSL does not disturb barrier integrity like 3-oxo-C12-HSL (40). Moreover, an AHL identified recently in the gut, 3-oxo-C12:2-HSL, exerts anti-inflammatory effects on Caco-2/TC7 cell line stimulated by interleukine-1β (IL-1β), as shown on IL-8-reduced secretion (13).

Some groups identified AHL as chemoattractant to neutrophils, as it is the case for 3-oxo-C12-HSL and 3-oxo-C10-HSL, but not C4-HSL. AHL attract neutrophils in a dose-dependent fashion, through actin remodeling and calcium mobilization (42, 43). Moreover, 3-oxo-C12-HSL exerts proapoptotic function on neutrophils, by targeting mitochondria and their calcium balance (43).

Macrophages are one of the most studied cell type regarding 3-oxo-C12-HSL impacts. Overall, the observed effects aim at decreasing the inflammatory response, allowing the set-up of a chronic *P. aeruginosa* infection. The effects are various, from cell-volume increase through water flux (44), unfold protein response (UPR) (45) to apoptosis (46) and also on immune functions. Indeed, *P. aeruginosa* 3-oxo-C12-HSL exerts anti-inflammatory responses in macrophages, as was reported by Glucksam-Galnoy et al. on RAW264.7 murine macrophages, in a dose-dependent fashion. Notably, a decrease in tumor necrosis factor-α production and increase in IL-10 secretion was observed (47). As mentioned earlier, when macrophages are in a proinflammatory context, 3-oxo-C12-HSL can modulate the NF-κB pathway resulting in a decrease in the expression of proinflammatory cytokines such as tumor necrosis factor-α (TNFα), regulated upon activation, normal T cell expressed, and secreted (RANTES), or monocyte chemoattractant protein-1 (MCP-1) (48). The involvement of MAPK p38 has also been reported several times (21, 47, 49) but needs further studies to determine its role in the signaling. In addition, it has been shown that macrophages in the presence of 3-oxo-C12-HSL have a higher phagocytic activity (49, 50), and those effects are abolished when MAPK p38 is inhibited (49).

Dendritic cells (DCs) stimulated by lipopolysaccharides and in the presence of 3-oxo-C12-HSL show a decrease in their proinflammatory cytokine such as IL-12 and interferon-γ (IFNγ) (51–53). However, reports on anti-inflammatory IL-10 are contradictory. In both studies, the cells were stimulated by lipopolysaccharides and exposed to the same dose of 3-oxo-C12-HSL; one team reported an increase in IL-10 secretion by human DCs (53), while another group did not see any change in IL-10 production by mouse DCs (52). By modulating DCs activation through its QS molecules, *P. aeruginosa* suppress the adaptive immune response, favoring the establishment of chronic infection (52, 53). 3-oxo-C12-HSL has also proapoptotic effects on humans DCs, as is the case for several cell types (54).

AHL have cell-type-specific impacts, but overall, the effects tend to be anti-inflammatory. Besides, 3-oxo-C12-HSL effects on apoptosis are dependent on the cell types, as multiple studies do not report toxicity on differentiated epithelial cells or fibroblasts (31, 55, 56). The effects of 3-oxo-C12-HSL on immune cells are compiled in the Table S1. These pathogen-related AHL also display multiple disruptions on several cell functions from innate immune cells.

### Impact of AHL on Adaptive Immunity

Once again, most of the knowledge in that field relies on 3-oxo-C12-HSL from *P. aeruginosa*. It had been first shown that this AHL could inhibit T-cell proliferation (57). This was confirmed by later reports showing that the same AHL could inhibit the proliferation and function (cytokine production) of both mitogen-stimulated (11, 58) and antigen-stimulated (59) T lymphocytes and modulate antibody production by B lymphocytes (57, 58). Overall, AHL from *P. aeruginosa* tend to have a less effective antibody-mediated, rather than a more effective cell-mediated, adaptive immune response to the bacteria, and could thus facilitate persistence of the pathogen. A structure–activity relationship study of 3-oxo-C12-HSL indicated that, like QS activity, immune modulatory activity requires an intact HSL ring, L-configuration at the chiral center, and an acyl chain of 11–13 carbons (11). Moreover, 3-oxo-C12-HSL can rapidly induce apoptosis via mitochondrial pathway on Jurkat cell line (60) and can inhibit DCs and T-cell activation and proliferation, and downregulate the expression of costimulatory molecules on DCs (54, 61, 62). This results in shifting immune responses away from host-protective Th1 responses to pathogen-protective Th2 responses (62). More precisely, 3-oxo-C12-HSL is able to promote the induction of regulatory T cells such as CD4+CD25+Foxp3+ induced regulatory T cells and to enhance their IL-10 and transforming growth factor-β (TGFβ) production associated with reduced IFNγ and IL-12p70 production (53). At the molecular level, 3-oxo-C12-HSL prevents human DCs maturation by blocking the upregulation of surface molecules, including CD11c, HLA-DR, CD40, and CD80, and DCs switched to an interleukin IL-10 (high), IL-12p70 (low) phenotype (53).

In addition, it has been reported that 3-oxo-C12-HSL increases the expression of Toll-like receptor-2 (TLR2), in a dose-dependent fashion, in lymphocytes from peripheral blood (63). Those observations are interesting because it has been shown that this AHL signaling does not rely on TLR recognition (21).

Taking together, there are limited observations showing T-cell inhibition and Treg induction by one AHL from a pathogen. It is crucial to look at the effect of other natural AHL especially those coming from commensal gut bacteria.

### AHL Within Intestinal Communities

#### Clues on the Presence of AHL in the Gut

The existence of AHL in the gut has been subject to questioning. An article from 2013 entitled “Are There Acyl-Homoserine Lactones within Mammalian Intestines?” examine the question (64). Mammalian pathogens such as *P. aeruginosa* or *Yersinia enterocolitica* are well known for their ability to produce AHL, but what about commensal bacteria? As mentioned by the authors, previous studies relied on the use of LuxR-based biosensors, where the detection limit may be too high to detect AHL from the gut, and there is thus a need for the development of new more sensitive tools (64). Moreover, the Human Microbiome Project, aiming at sequencing and analyzing the microbiome of several cohorts, gives clues about the presence of AHL in the intestines. Indeed, the LuxI/LuxR homolog has been found in three strains from the gastrointestinal tract: *Hafnia alvei, Edwardsiella tarda*, and *Ralstonia* sp. strain 5_7_47FAA (64). In addition, bacteria like *E. coli, Enterobacter*, or *Klebsiella* are part of the normal gut microbiota and express, as mentioned above, the receptor SdiA and therefore can sense AHL (17). One must highlight the fragility of small molecules like AHL. Indeed, they are pH sensitive and can also be inactivated by the presence of ont only host lactonases like PON but also bacterial enzymes. Those degradations can decrease the concentration of the molecules, making it harder for biosensors to reach the threshold detection (64). Moreover, it is also possible that auto-inducers are present in the mammalian gut, but with a yet unknown structure, like aryl-homoserine lactones, with an aromatic side chain instead of an acyl chain and thus not detectable by biosensors (64). It is also interesting to note that AHL have been found in the rumen of cattle by several teams (65, 66).

Our team investigated the question of AHL in the human gut, in the context of IBD. By using mass spectrometry, we were able to detect 14 different AHLs in the feces of IBD patients and healthy subject, and the distribution of the AHL were correlated to the disease state (13). Indeed, one of the AHL was prominent: 3-oxo-C12:2-HSL. This molecule was, until then, undescribed and carries two insaturations on its acyl chain. 3-Oxo-C12:2-HSL was highly decreased in fecal samples of IBD patients in flare (16%) compared to remission patients (37.5%) and to healthy subjects (64.5%) (13). The absence of this AHL was correlated with dysbiosis and a decrease in Firmicutes, considered as beneficial bacteria. In addition, 3-oxo-C12:2-HSL exerts anti-inflammatory properties on intestinal cell lines Caco-2/TC7 (13).

#### Toward the Use of AHL to Modulate Microbiota Composition and Gut Inflammation

**Figure 2:**
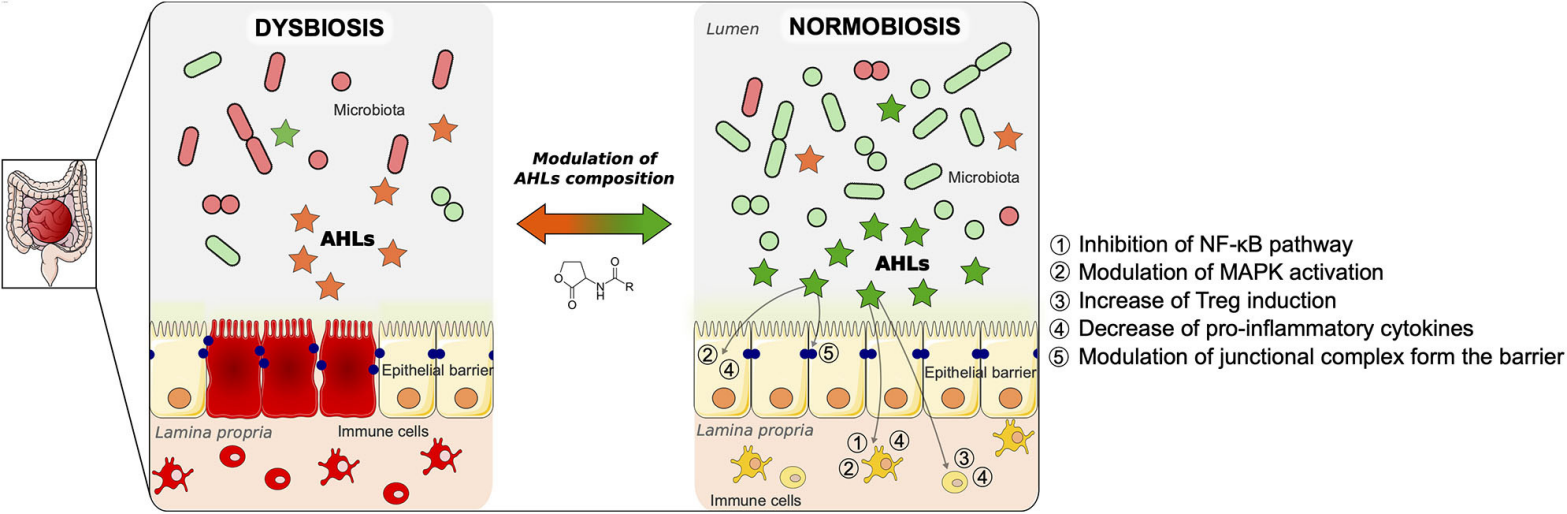
Proposed model of modulation of gut mucosa inflammation by *N*-acyl-homoserine lactone (AHL)-driven quorum sensing and associated cellular pathways. Inflammatory bowel disease (IBD) is the result of multiple factors. It involves an imbalance of the microbiota (dysbiosis), an alteration of the epithelial barrier, as well as an uncontrolled inflammation in the gut mucosa, as described on the left panel. As a result of the dysbiosis, the AHL composition is changed compared to a physiological state. During normobiosis (right panel), when the bacterial communities are balanced, the AHL profile is modulated compared to a disease state. Beyond reshaping bacterial composition, AHL can modulate the inflammatory state of gut mucosa as well as restore epithelial barrier integrity. The pathways involved in those effects are listed on the right panel of the figure. We propose a strategy to control gut inflammation by modulating AHL composition using natural or synthetic AHL.

The study of QS molecules is mainly done on single species bacteria or *in vitro* cells. As emphasized by Karina Bivar Xavier, QS is also an interspecies communication network, and more studies on the impact of QS on multi-communities ecosystems should be done (67). *In vivo* studies are crucial to understanding relationships in an environment as complex as the intestine. Kumari et al. have developed a whole-cell sensing systems for the detection of AHL and have shown that these signaling molecules detected in saliva and stool may be potential non-invasive biomarkers of gastrointestinal inflammatory disease (68). To emphasize this point, given that AHL profile relies on bacterial dynamics, it could be considered as an indicator of dysbiosis, opening new perspectives in managing chronical diseases such as IBD. Understanding the interactions between bacteria in high concentrations and high diversity can help us decipher what species are most beneficial to mammalian gut. To note, another QS type of molecule, AI-2, has been used to modulate gut microbiota composition and dysbiosis (69). As illustrated in Figure 2, AHLs remain good candidates in the strategy to use natural molecules from QS to modulate microbiota composition and gut inflammation.

## Concluding Remarks

Gut microbiota mutually interacts with coevolved host epithelial and immune cells in a beneficial reciprocal relationship. QS signaling of bacteria probably contributes substantially to establishing symbiotic interactions in some cross-kingdom interactive dynamics. In IBD, where, host–microbiota interactions drive inflammatory response, looking at quorum sensing changes and impact on immunity appears as a completely novel and original approach. The mechanisms, including the regulation of synthesis and degradation of these diffusible signaling molecules are still not completely understood. However, the discovery of the general importance of auto-inducer signaling molecules involved in quorum or efficiency sensing of bacteria and the dynamic and immune responses by eukaryotes toward them opens up a new field of pathophysiology.

## Author Contributions

GC and PS drew the outline of the manuscript. GC, J-PG, and PS wrote and proofread the manuscript. All authors contributed to the article and approved the submitted version.

## Conflict of Interest

The authors declare that the research was conducted in the absence of any commercial or financial relationships that could be construed as a potential conflict of interest.

## Abbreviations

AHL: *N*-acyl-homoserine lactones
3-oxo-C12-HSL: *N*-(3-oxododecanoyl)-l-homoserine lactone
AI: auto-inducer
C4-HSL: *N*-butyryl-homoserine lactone
DCs: dendritic cells
IBD: inflammatory bowel disease
IFNγ: interferon-γ
IL-10: interleukine 10
IQGAP1: IQ motif containing GTPase-activating protein 1
MAPK: mitogen-activated protein kinase
PON: paraoxonase
PPAR: peroxisome proliferator-activated receptor
PRR: pattern recognition receptor
QS: quorum sensing
TGFβ: transforming growth factor-β
TLR-2: Toll-like receptor-2
TNFα: tumor necrosis factor-α
UPR: unfolded protein response.

## References

[1] Maloy KJ Powrie F. Intestinal homeostasis and its breakdown in inflammatory bowel disease. Nature. (2011) 474:298–306. 10.1038/nature10208 21677746

[2] Bassler BL Wright M Silverman MR. Multiple signalling systems controlling expression of luminescence in *Vibrio harveyi* : sequence and function of genes encoding a second sensory pathway. Mol Microbiol. (1994) 13:273–86. 10.1111/j.1365-2958.1994.tb00422.x 7984107

[3] Abisado RG Benomar S Klaus JR Dandekar AA Chandler JR Bacterial quorum sensing and microbial community interactions. mBio. (2018) 9:14 10.1128/mBio.02331-17 PMC5964356 29789364

[4] Mathesius U Mulders S Gao M Teplitski M Caetano-Anolles G Rolfe BG. Extensive and specific responses of a eukaryote to bacterial quorum-sensing signals. Proc Natl Acad Sci USA. (2003) 100:1444–9. 10.1073/pnas.262672599 12511600 PMC298792

[5] Schikora A Schenk ST Hartmann A. Beneficial effects of bacteria-plant communication based on quorum sensing molecules of the N -acyl homoserine lactone group. Plant Mol Biol. (2016) 90:605–12. 10.1007/s11103-016-0457-8 26898296

[6] Liu F Zhao Q Jia Z Song C Huang Y Ma H. N-3-oxo-octanoyl-homoserine lactone-mediated priming of resistance to *Pseudomonas syringae* requires the salicylic acid signaling pathway in *Arabidopsis thaliana*. BMC Plant Biol. (2020) 20:38. 10.21203/rs.2.11827/v2 31992205 PMC6986161

[7] Hu Z Shao S Zheng C Sun Z Shi J Yu J. Induction of systemic resistance in tomato against *Botrytis cinerea* by N-decanoyl-homoserine lactone via jasmonic acid signaling. Planta. (2018) 247:1217–27. 10.1007/s00425-018-2860-7 29445868

[8] Nealson KH Platt T Hastings JW. Cellular control of the synthesis and activity of the bacterial luminescent system1. J Bacteriol. (1970) 104:313–22. 10.1128/JB.104.1.313-322.1970 5473898 PMC248216

[9] Fuqua WC Winans SC Greenberg EP. Quorum sensing in bacteria: the LuxR-LuxI family of cell density-responsive transcriptional regulators. J Bacteriol. (1994) 176:269–75. 10.1128/JB.176.2.269-275.1994 8288518 PMC205046

[10] Holm A Vikström E. Quorum sensing communication between bacteria and human cells: signals, targets, and functions. Front Plant Sci. (2014) 5:309. 10.3389/fpls.2014.00309 25018766 PMC4071818

[11] Chhabra SR Harty C Hooi DSW Daykin M Williams P Telford G. Synthetic analogues of the bacterial signal (quorum sensing) molecule N -(3-Oxododecanoyl)- l -homoserine lactone as immune modulators. J Med Chem. (2003) 46:97–104. 10.1021/jm020909n 12502363

[12] Whiteley M Diggle SP Greenberg EP. Progress in and promise of bacterial quorum sensing research. Nature. (2017) 551:313–20. 10.1038/nature24624 29144467 PMC5870893

[13] Landman C Grill J-P Mallet J-M Marteau P Humbert L Balc'h EL. Inter-kingdom effect on epithelial cells of the N-Acyl homoserine lactone 3-oxo-C12:2, a major quorum-sensing molecule from gut microbiota. PLoS ONE. (2018) 13:e0202587. 10.1371/journal.pone.0202587 30157234 PMC6114859

[14] Parsek MR Val DL Hanzelka BL Cronan JE Greenberg EP. Acyl homoserine-lactone quorum-sensing signal generation. Proc Natl Acad Sci USA. (1999) 96:4360–5. 10.1073/pnas.96.8.4360 10200267 PMC16337

[15] Prescott RD Decho AW. Flexibility and adaptability of quorum sensing in nature. Trends Microbiol. (2020) 28:436–44. 10.1016/j.tim.2019.12.004 32001099 PMC7526683

[16] Hudaiberdiev S Choudhary KS Vera Alvarez R Gelencsér Z Ligeti B Lamba D. Census of solo LuxR genes in prokaryotic genomes. Front Cell Infect Microbiol. (2015) 5:20. 10.3389/fcimb.2015.00020 25815274 PMC4357305

[17] Tobias NJ Brehm J Kresovic D Brameyer S Bode HB Heermann R. New vocabulary for bacterial communication. ChemBioChem. (2020) 21:759–68. 10.1002/cbic.201900580 31709676 PMC7154725

[18] Shiner EK Rumbaugh KP Williams SC. Interkingdom signaling: deciphering the language of acyl homoserine lactones. FEMS Microbiol Rev. (2005) 29:935–47. 10.1016/j.femsre.2005.03.001 16219513

[19] Ritchie AJ Whittall C Lazenby JJ Chhabra SR Pritchard DI Cooley MA The immunomodulatory *Pseudomonas aeruginosa* signalling molecule N -(3-oxododecanoyl)- l -homoserine lactone enters mammalian cells in an unregulated fashion. Immunol Cell Biol. (2007) 85:596–602. 10.1038/sj.icb.7100090 17607318

[20] Williams P Cámara M. Quorum sensing and environmental adaptation in *Pseudomonas aeruginosa*: a tale of regulatory networks and multifunctional signal molecules. Curr Opin Microbiol. (2009) 12:182–91. 10.1016/j.mib.2009.01.005 19249239

[21] Kravchenko VV Kaufmann GF Mathison JC Scott DA Katz AZ Wood MR. N -(3-Oxo-acyl)homoserine lactones signal cell activation through a mechanism distinct from the canonical pathogen-associated molecular pattern recognition receptor pathways. J Biol Chem. (2006) 281:28822–30. 10.1074/jbc.M606613200 16893899

[22] Li L Hooi D Chhabra SR Pritchard D Shaw PE. Bacterial N-acylhomoserine lactone-induced apoptosis in breast carcinoma cells correlated with down-modulation of STAT3. Oncogene. (2004) 23:4894–902. 10.1038/sj.onc.1207612 15064716

[23] Jahoor A Patel R Bryan A Do C Krier J Watters C. Peroxisome proliferator-activated receptors mediate host cell proinflammatory responses to *Pseudomonas aeruginosa* autoinducer. J Bacteriol. (2008) 190:4408–15. 10.1128/JB.01444-07 18178738 PMC2446782

[24] Cooley MA Whittall C Rolph MS. Pseudomonas signal molecule 3-oxo-C12-homoserine lactone interferes with binding of rosiglitazone to human PPARγ. Microbes Infect. (2010) 12:231–7. 10.1016/j.micinf.2009.12.009 20074659

[25] Behrens M Meyerhof W. Gustatory and extragustatory functions of mammalian taste receptors. Physiol Behav. (2011) 105:4–13. 10.1016/j.physbeh.2011.02.010 21324331

[26] Rozengurt E Taste receptors in the gastrointestinal tract. I. Bitter taste receptors and alpha-gustducin in the mammalian gut. Am J Physiol Gastrointest Liver Physiol. (2006) 291:G171–7. 10.1152/ajpgi.00073.2006 16710053

[27] Carrai M Steinke V Vodicka P Pardini B Rahner N Holinski-Feder E. Association between TAS2R38 gene polymorphisms and colorectal cancer risk: a case-control study in two independent populations of Caucasian origin. PLoS ONE. (2011) 6:e20464. 10.1371/journal.pone.0020464 21674048 PMC3107225

[28] Lee RJ Xiong G Kofonow JM Chen B Lysenko A Jiang P. T2R38 taste receptor polymorphisms underlie susceptibility to upper respiratory infection. J Clin Invest. (2012) 122:4145–59. 10.1172/JCI64240 23041624 PMC3484455

[29] Gaida MM Mayer C Dapunt U Stegmaier S Schirmacher P Wabnitz GH. Expression of the bitter receptor T2R38 in pancreatic cancer: localization in lipid droplets and activation by a bacteria-derived quorum-sensing molecule. Oncotarget. (2016) 7:12623–32. 10.18632/oncotarget.7206 26862855 PMC4914309

[30] Maurer S Wabnitz GH Kahle NA Stegmaier S Prior B Giese T. Tasting *Pseudomonas aeruginosa* biofilms: human neutrophils express the bitter receptor T2R38 as sensor for the quorum sensing molecule N-(3-oxododecanoyl)-l-homoserine lactone. Front Immunol. (2015) 6:369. 10.3389/fimmu.2015.00369 26257736 PMC4513437

[31] Karlsson T Turkina MV Yakymenko O Magnusson K-E Vikström E. The *Pseudomonas aeruginosa* N-acylhomoserine lactone quorum sensing molecules target IQGAP1 and modulate epithelial cell migration. PLoS Pathog. (2012) 8:e1002953. 10.1371/journal.ppat.1002953 23071436 PMC3469656

[32] Tanos BE Yeaman C Rodriguez-Boulan E. An emerging role for IQGAP1 in tight junction control. Small GTPases. (2018) 9:375–83. 10.1080/21541248.2016.1244440 27880081 PMC5997139

[33] Bedi B Maurice NM Ciavatta VT Lynn KS Yuan Z Molina SA. Peroxisome proliferator-activated receptor-γ agonists attenuate biofilm formation by *Pseudomonas aeruginosa*. FASEB J. (2017) 31:3608–21. 10.1096/fj.201700075R 28442545 PMC5503711

[34] Cohen LJ Esterhazy D Kim S-H Lemetre C Aguilar RR Gordon EA. Commensal bacteria make GPCR ligands that mimic human signalling molecules. Nature. (2017) 549:48–53. 10.1038/nature23874 28854168 PMC5777231

[35] Grandclément C Tannières M Moréra S Dessaux Y Faure D. Quorum quenching: role in nature and applied developments. FEMS Microbiol Rev. (2016) 40:86–116. 10.1093/femsre/fuv038 26432822

[36] Karban A Hartman C Eliakim R Waterman M Nesher S Barnett-Griness O. Paraoxonase (PON)1 192R allele carriage is associated with reduced risk of inflammatory bowel disease. Dig Dis Sci. (2007) 52:2707–15. 10.1007/s10620-006-9700-5 17436100

[37] Vikström E Tafazoli F Magnusson K-E. Pseudomonas aeruginosa quorum sensing molecule N -(3 oxododecanoyl)- l -homoserine lactone disrupts epithelial barrier integrity of Caco-2 cells. FEBS Lett. (2006) 580:6921–8. 10.1016/j.febslet.2006.11.057 17157842

[38] Vikström E Bui L Konradsson P Magnusson K-E. The junctional integrity of epithelial cells is modulated by *Pseudomonas aeruginosa* quorum sensing molecule through phosphorylation-dependent mechanisms. Exp Cell Res. (2009) 315:313–26. 10.1016/j.yexcr.2008.10.044 19038248

[39] Vikström E Bui L Konradsson P Magnusson K-E. Role of calcium signalling and phosphorylations in disruption of the epithelial junctions by *Pseudomonas aeruginosa* quorum sensing molecule. Eur J Cell Biol. (2010) 89:584–97. 10.1016/j.ejcb.2010.03.002 20434232

[40] Eum SY Jaraki D Bertrand L Andras IE Toborek M. Disruption of epithelial barrier by quorum-sensing N-3-(oxododecanoyl)-homoserine lactone is mediated by matrix metalloproteinases. AJP Gastrointest Liver Physiol. (2014) 306:G992–1001. 10.1152/ajpgi.00016.2014 24742991 PMC4042118

[41] Schwarzer C Wong S Shi J Matthes E Illek B Ianowski JP. Pseudomonas aeruginosa homoserine lactone activates store-operated cAMP and cystic fibrosis transmembrane regulator-dependent Cl ^−^ secretion by human airway epithelia. J Biol Chem. (2010) 285:34850–63. 10.1074/jbc.M110.167668 20739289 PMC2966100

[42] Karlsson T Musse F Magnusson K-E Vikstrom E. N-Acylhomoserine lactones are potent neutrophil chemoattractants that act via calcium mobilization and actin remodeling. J Leukoc Biol. (2012) 91:15–26. 10.1189/jlb.0111034 21807742

[43] Singh PK Yadav VK Kalia M Sharma D Pandey D Agarwal V. *Pseudomonas aeruginosa* quorum-sensing molecule N-(3-oxo-dodecanoyl)-l-homoserine lactone triggers mitochondrial dysfunction and apoptosis in neutrophils through calcium signaling. Med Microbiol Immunol. (2019) 208:855–68. 10.1007/s00430-019-00631-8 31377870

[44] Holm A Magnusson K-E Vikström E. *Pseudomonas aeruginosa* N-3-oxo-dodecanoyl-homoserine lactone elicits changes in cell volume, morphology, and AQP9 characteristics in macrophages. Front Cell Infect Microbiol. (2016) 6:32. 10.3389/fcimb.2016.00032 27047801 PMC4805602

[45] Zhang J Gong F Li L Zhao M Song J. *Pseudomonas aeruginosa* quorum-sensing molecule N-(3-oxododecanoyl) homoserine lactone attenuates lipopolysaccharide-induced inflammation by activating the unfolded protein response. Biomed Rep. (2014) 2:233–8. 10.3892/br.2014.225 24649102 PMC3917758

[46] Tateda K Ishii Y Horikawa M Matsumoto T Miyairi S Pechere JC. The *Pseudomonas aeruginosa* autoinducer N-3-oxododecanoyl homoserine lactone accelerates apoptosis in macrophages and neutrophils. Infect Immun. (2003) 71:5785–93. 10.1128/IAI.71.10.5785-5793.2003 14500500 PMC201082

[47] Glucksam-Galnoy Y Sananes R Silberstein N Krief P Kravchenko VV Meijler MM. The bacterial quorum-sensing signal molecule N-3-oxo-dodecanoyl-L-homoserine lactone reciprocally modulates pro- and anti-inflammatory cytokines in activated macrophages. J Immunol. (2013) 191:337–44. 10.4049/jimmunol.1300368 23720811 PMC3691282

[48] Kravchenko VV Kaufmann GF Mathison JC Scott DA Katz AZ Grauer DC. Modulation of gene expression via disruption of NF- B signaling by a bacterial small molecule. Science. (2008) 321:259–63. 10.1126/science.1156499 18566250

[49] Vikström E Magnusson K-E Pivoriunas A. The *Pseudomonas aeruginosa* quorum-sensing molecule N-(3-oxododecanoyl)-l-homoserine lactone stimulates phagocytic activity in human macrophages through the p38 MAPK pathway. Microbes Infect. (2005) 7:1512–8. 10.1016/j.micinf.2005.05.012 16039899

[50] Holm A Karlsson T Vikström E. *Pseudomonas aeruginosa* lasI/rhlI quorum sensing genes promote phagocytosis and aquaporin 9 redistribution to the leading and trailing regions in macrophages. Front Microbiol. (2015) 6:915. 10.3389/fmicb.2015.00915 26388857 PMC4558532

[51] Guo J Yoshida K Ikegame M Okamura H. Quorum sensing molecule N-(3-oxododecanoyl)-l-homoserine lactone: an all-rounder in mammalian cell modification. J Oral Biosci. (2020) 62:16–29. 10.1016/j.job.2020.01.001 31982630

[52] Skindersoe ME Zeuthen LH Brix S Fink LN Lazenby J Whittall C. *Pseudomonas aeruginosa* quorum-sensing signal molecules interfere with dendritic cell-induced T-cell proliferation. FEMS Immunol Med Microbiol. (2009) 55:335–45. 10.1111/j.1574-695X.2008.00533.x 19187218

[53] Li Y Zhou H Zhang Y Chen C Huang B Qu P. N−3-(oxododecanoyl)-L-homoserine lactone promotes the induction of regulatory T-cells by preventing human dendritic cell maturation. Exp Biol Med. (2015) 240:896–903. 10.1177/1535370214564742 25749498 PMC4935398

[54] Boontham P Robins A Chandran P Pritchard D Cámara M Williams P. Significant immunomodulatory effects of *Pseudomonas aeruginosa* quorum-sensing signal molecules: possible link in human sepsis. Clin Sci. (2008) 115:343–51. 10.1042/CS20080018 18363571

[55] Josephson H Ntzouni M Skoglund C Linder S Turkina MV Vikström E. *Pseudomonas aeruginosa* N-3-oxo-dodecanoyl-homoserine lactone impacts mitochondrial networks morphology, energetics, and proteome in host cells. Front Microbiol. (2020) 11:1069. 10.3389/fmicb.2020.01069 32523583 PMC7261938

[56] Losa D Köhler T Bacchetta M Saab JB Frieden M van Delden C. Airway epithelial cell integrity protects from cytotoxicity of *Pseudomonas aeruginosa* quorum-sensing signals. Am J Respir Cell Mol Biol. (2015) 53:265–75. 10.1165/rcmb.2014-0405OC 25562674

[57] Telford G Wheeler D Williams P Tomkins PT Appleby P Sewell H. The *Pseudomonas aeruginosa* quorum-sensing signal molecule N-(3-oxododecanoyl)-l-homoserine lactone has immunomodulatory activity. Infect Immun. (1998) 66:36–42. 10.1128/IAI.66.1.36-42.1998 9423836 PMC107855

[58] Ritchie AJ Yam AOW Tanabe KM Rice SA Cooley MA. Modification of *in vivo* and *in vitro* T- and B-cell-mediated immune responses by the *Pseudomonas aeruginosa* quorum-sensing molecule N-(3-oxododecanoyl)-l-homoserine lactone. Infect Immun. (2003) 71:4421–31. 10.1128/IAI.71.8.4421-4431.2003 12874321 PMC165988

[59] Ritchie AJ Jansson A Stallberg J Nilsson P Lysaght P Cooley MA. The *Pseudomonas aeruginosa* quorum-sensing molecule N-3-(oxododecanoyl)-l-homoserine lactone inhibits T-cell differentiation and cytokine production by a mechanism involving an early step in T-cell activation. Infect Immun. (2005) 73:1648–55. 10.1128/IAI.73.3.1648-1655.2005 15731065 PMC1064928

[60] Jacobi CA Schiffner F Henkel M Waibel M Stork B Daubrawa M. Effects of bacterial N-acyl homoserine lactones on human Jurkat T lymphocytes-OdDHL induces apoptosis via the mitochondrial pathway. Int J Med Microbiol. (2009) 299:509–19. 10.1016/j.ijmm.2009.03.005 19464950

[61] Hooi DSW Bycroft BW Chhabra SR Williams P Pritchard DI. Differential immune modulatory activity of pseudomonas aeruginosa quorum-sensing signal molecules. Infect Immun. (2004) 72:6463–70. 10.1128/IAI.72.11.6463-6470.2004 15501777 PMC522992

[62] Gupta RK Chhibber S Harjai K. Acyl homoserine lactones from culture supernatants of *Pseudomonas aeruginosa* accelerate host immunomodulation. PLoS ONE. (2011) 6:e20860. 10.1371/journal.pone.0020860 21698201 PMC3116856

[63] Bao L Yu J Zhong H Huang D Lu Q. Expression of toll-like receptors in T lymphocytes stimulated with N -(3-oxododecanoyl)-L-homoserine lactone from *Pseudomonas aeruginosa*. APMIS. (2017) 125:553–7. 10.1111/apm.12690 28418096

[64] Swearingen MC Sabag-Daigle A Ahmer BMM. Are there acyl-homoserine lactones within mammalian intestines? J Bacteriol. (2013) 195:173–9. 10.1128/JB.01341-12 23144246 PMC3553842

[65] Erickson DL Nsereko VL Morgavi DP Selinger LB Rode LM Beauchemin KA. Evidence of quorum sensing in the rumen ecosystem: detection of N -acyl homoserine lactone autoinducers in ruminal contents. Can J Microbiol. (2002) 48:374–8. 10.1139/w02-022 12030712

[66] Won M-Y Oyama LB Courtney SJ Creevey CJ Huws SA. Can rumen bacteria communicate to each other? Microbiome. (2020) 8:23. 10.1186/s40168-020-00796-y 32085816 PMC7035670

[67] Bivar Xavier K. Bacterial interspecies quorum sensing in the mammalian gut microbiota. C R Biol. (2018) 341:297–9. 10.1016/j.crvi.2018.03.006 29631889

[68] Kumari A Pasini P Deo SK Flomenhoft D Shashidhar H Daunert S. Biosensing systems for the detection of bacterial quorum signaling molecules. Anal Chem. (2006) 78:7603–9. 10.1021/ac061421n 17105149

[69] Thompson JA Oliveira RA Djukovic A Ubeda C Xavier KB. Manipulation of the quorum sensing signal AI-2 affects the antibiotic-treated gut microbiota. Cell Rep. (2015) 10:1861–71. 10.1016/j.celrep.2015.02.049 25801025

