# Supplementary material for: Impact of N-Acyl-Homoserine Lactones, Quorum Sensing Molecules, on Gut Immunity
Source: Front Immunol. 2020 Aug 28;11:1827. doi: 10.3389/fimmu.2020.01827 (PMC7484616; doi:10.3389/fimmu.2020.01827)
Supplement: Supplementary file 1 [file Data_Sheet_1.pdf]

Supplementary material :

Table 1 : Summary table of the effects of the Acyl-Homoserine Lactone 3-oxo-C12-HSL exerted on immune cells

**Cell type**	**Effects of 3-oxo-C12-HSL**	**Reference**
**Innate immune cells**		
Intestinal Epithelial Cells	- barrier integrity altered<br>- migration modulation<br>- impact on cell viability<br>-	- (1–4)<br>- (5)<br>- (6)
Macrophages	- anti-inflammatory properties on IL-12 and TNFα<br>- Pro-apoptotic<br>- Increased phagocytosis<br>- NF-κB inhibition<br>- Dose-dependent anti-inflammatory effects<br>- Involvement of MAPK p38 in signaling<br>- Activation of UPR<br>- Change in cell volume and shape	- (7)<br>- (8)<br>- (9)<br>- (10)<br>- (11)<br>- (9,12,11)<br>- (13)<br>- (14)
Dendritic cells	- pro-apoptotic effects<br>- No effects on IL-10 secretion<br>- Increase IL-10 production<br>- Decrease of IL-12 secretion<br>- increase of Treg induction	- (15)<br>- (16)<br>- (17)<br>- (16,17)<br>- (17)
Neutrophils	- chemoattractant<br>- activation of MAPK<br>- pro-apoptotic	- (5,18)<br>- (19)<br>- (8,20)
**Adaptive immune cells**		
T cells	- Inhibition of proliferation and activation<br>- Activation<br>- Reduction of secretion of IL-4 and d'IFNγ<br>- Induction of apoptosis via the mitochondria pathway<br>- Induction of regulatory T cells	- (7,15,21,22)<br>- (23)<br>- (24)<br>- (25)<br>- (17)
B cells	- Modulation of immunoglobulin production	- (7,26)

1. Vikström E, Tafazoli F, Magnusson K-E. *Pseudomonas aeruginosa* quorum sensing molecule *N* -(3 oxododecanoyl)- L -homoserine lactone disrupts epithelial barrier integrity of Caco-2 cells. FEBS Letters. 22 déc 2006;580(30):6921-8.

2. Vikström E, Bui L, Konradsson P, Magnusson K-E. The junctional integrity of epithelial cells is modulated by Pseudomonas aeruginosa quorum sensing molecule through phosphorylation-dependent mechanisms. Experimental Cell Research. janv 2009;315(2):313-26.

3. Vikström E, Bui L, Konradsson P, Magnusson K-E. Role of calcium signalling and phosphorylations in disruption of the epithelial junctions by Pseudomonas aeruginosa quorum sensing molecule. European Journal of Cell Biology. août 2010;89(8):584-97.

4. Eum SY, Jaraki D, Bertrand L, Andras IE, Toborek M. Disruption of epithelial barrier by quorum-sensing N-3-(oxododecanoyl)-homoserine lactone is mediated by matrix metalloproteinases. AJP: Gastrointestinal and Liver Physiology. 1 juin 2014;306(11):G992-1001.

5. Karlsson T, Musse F, Magnusson K-E, Vikstrom E. N-Acylhomoserine lactones are potent neutrophil chemoattractants that act via calcium mobilization and actin remodeling. Journal of Leukocyte Biology. 1 janv 2012;91(1):15-26.

6. Tao S, Sun Q, Cai L, Geng Y, Hua C, Ni Y, et al. Caspase-1-dependent mechanism mediating the harmful impacts of the quorum-sensing molecule N-(3-oxo-dodecanoyl)-l-homoserine lactone on the intestinal cells. Journal of Cellular Physiology [Internet]. [cité 3 déc 2018];0(0). Disponible sur: https://onlinelibrary.wiley.com/doi/abs/10.1002/jcp.27132

7. Telford G, Wheeler D, Williams P, Tomkins PT, Appleby P, Sewell H, et al. The Pseudomonas aeruginosaQuorum-Sensing Signal MoleculeN-(3-Oxododecanoyl)-l-Homoserine Lactone Has Immunomodulatory Activity. Infection and Immunity. 1998;66(1):36-42.

8. Tateda K, Ishii Y, Horikawa M, Matsumoto T, Miyairi S, Pechere JC, et al. The Pseudomonas aeruginosa Autoinducer N-3-Oxododecanoyl Homoserine Lactone Accelerates Apoptosis in Macrophages and Neutrophils. Infection and Immunity. 1 oct 2003;71(10):5785-93.

9. Vikström E, Magnusson K-E, Pivoriūnas A. The Pseudomonas aeruginosa quorum-sensing molecule N-(3-oxododecanoyl)-l-homoserine lactone stimulates phagocytic activity in human macrophages through the p38 MAPK pathway. Microbes and Infection. déc 2005;7(15):1512-8.

10. Kravchenko VV, Kaufmann GF, Mathison JC, Scott DA, Katz AZ, Grauer DC, et al. Modulation of Gene Expression via Disruption of NF- B Signaling by a Bacterial Small Molecule. Science. 11 juill 2008;321(5886):259-63.

11. Glucksam-Galnoy Y, Sananes R, Silberstein N, Krief P, Kravchenko VV, Meijler MM, et al. The Bacterial Quorum-Sensing Signal Molecule N-3-Oxo-Dodecanoyl-L-Homoserine Lactone Reciprocally Modulates Pro- and Anti-Inflammatory Cytokines in Activated Macrophages. The Journal of Immunology. 1 juill 2013;191(1):337-44.

12. Kravchenko VV, Kaufmann GF, Mathison JC, Scott DA, Katz AZ, Wood MR, et al. *N*-(3-Oxo-acyl)homoserine Lactones Signal Cell Activation through a Mechanism distinct from the Canonical Pathogen-associated Molecular Pattern Recognition Receptor Pathways. Journal of Biological Chemistry. 29 sept 2006;281(39):28822-30.

13. Zhang J, Gong F, Li L, Zhao M, Song J. Pseudomonas aeruginosa quorum-sensing molecule N-(3-oxododecanoyl) homoserine lactone attenuates lipopolysaccharide-induced inflammation by activating the unfolded protein response. Biomedical Reports. mars 2014;2(2):233-8.

14. Holm A, Magnusson K-E, Vikström E. Pseudomonas aeruginosa N-3-oxo-dodecanoyl-homoserine Lactone Elicits Changes in Cell Volume, Morphology, and AQP9 Characteristics in Macrophages. Frontiers in Cellular and Infection Microbiology [Internet]. 24 mars 2016 [cité 9 mars 2018];6. Disponible sur: http://journal.frontiersin.org/Article/10.3389/fcimb.2016.00032/abstract

15. Boontham P, Robins A, Chandran P, Pritchard D, Cámara M, Williams P, et al. Significant immunomodulatory effects of Pseudomonas aeruginosa quorum-sensing signal molecules: possible link in human sepsis. Clinical Science. 1 déc 2008;115(11):343-51.

16. Skindersoe ME, Zeuthen LH, Brix S, Fink LN, Lazenby J, Whittall C, et al. *Pseudomonas aeruginosa* quorum-sensing signal molecules interfere with dendritic cell-induced T-cell proliferation. FEMS Immunol Med Microbiol. avr 2009;55(3):335-45.

17. Li Y, Zhou H, Zhang Y, Chen C, Huang B, Qu P, et al. *N*-3-(oxododecanoyl)-L-homoserine lactone promotes the induction of regulatory T-cells by preventing human dendritic cell maturation. Exp Biol Med (Maywood). juill 2015;240(7):896-903.

18. Zimmermann S, Wagner C, Muller W, Brenner-Weiss G, Hug F, Prior B, et al. Induction of Neutrophil Chemotaxis by the Quorum-Sensing Molecule N-(3-Oxododecanoyl)-l-Homoserine Lactone. INFECT IMMUN. 2006;74:6.

19. Kahle NA, Brenner-Weiss G, Overhage J, Obst U, Hänsch GM. Bacterial quorum sensing molecule induces chemotaxis of human neutrophils via induction of p38 and leukocyte specific protein 1 (LSP1). Immunobiology. févr 2013;218(2):145-51.

20. Singh PK, Yadav VK, Kalia M, Sharma D, Pandey D, Agarwal V. Pseudomonas aeruginosa quorum-sensing molecule N-(3-oxo-dodecanoyl)-l-homoserine lactone triggers mitochondrial dysfunction and apoptosis in neutrophils through calcium signaling. Med Microbiol Immunol [Internet]. 3 août 2019 [cité 19 août 2019]; Disponible sur: http://link.springer.com/10.1007/s00430-019-00631-8

21. Hooi DSW, Bycroft BW, Chhabra SR, Williams P, Pritchard DI. Differential Immune Modulatory Activity of Pseudomonas aeruginosa Quorum-Sensing Signal Molecules. Infection and Immunity. 1 nov 2004;72(11):6463-70.

22. Gupta RK, Chhibber S, Harjai K. Acyl Homoserine Lactones from Culture Supernatants of Pseudomonas aeruginosa Accelerate Host Immunomodulation. DeLeo FR, éditeur. PLoS ONE. 16 juin 2011;6(6):e20860.

23. Smith RS, Kelly R, Iglewski BH, Phipps RP. The Pseudomonas Autoinducer N-(3-Oxododecanoyl) Homoserine Lactone Induces Cyclooxygenase-2 and Prostaglandin E2 Production in Human Lung Fibroblasts: Implications for Inflammation. The Journal of Immunology. 1 sept 2002;169(5):2636-42.

24. Ritchie AJ, Jansson A, Stallberg J, Nilsson P, Lysaght P, Cooley MA. The Pseudomonas aeruginosa Quorum-Sensing Molecule N-3-(Oxododecanoyl)-L-Homoserine Lactone Inhibits T-Cell Differentiation and Cytokine Production by a Mechanism Involving an Early Step in T-Cell Activation. Infection and Immunity. 1 mars 2005;73(3):1648-55.

25. Jacobi CA, Schiffner F, Henkel M, Waibel M, Stork B, Daubrawa M, et al. Effects of bacterial N-acyl homoserine lactones on human Jurkat T lymphocytes-OdDHL induces apoptosis via the mitochondrial pathway. International Journal of Medical Microbiology. nov 2009;299(7):509-19.

26. Ritchie AJ, Yam AOW, Tanabe KM, Rice SA, Cooley MA. Modification of In Vivo and In Vitro T- and B-Cell-Mediated Immune Responses by the Pseudomonas aeruginosa Quorum-Sensing Molecule N-(3-Oxododecanoyl)-L-Homoserine Lactone. Infection and Immunity. 1 août 2003;71(8):4421-31.
